# The pro-apoptotic Bcl-2 family member Harakiri (HRK) induces cell death in glioblastoma multiforme

**DOI:** 10.1038/s41420-019-0144-z

**Published:** 2019-02-08

**Authors:** Ezgi Kaya-Aksoy, Ahmet Cingoz, Filiz Senbabaoglu, Fidan Seker, Ilknur Sur-Erdem, Alisan Kayabolen, Tolga Lokumcu, Gizem Nur Sahin, Sercin Karahuseyinoglu, Tugba Bagci-Onder

**Affiliations:** 1 Brain Cancer Research and Therapy Laboratory, Koç University School of Medicine, Istanbul, Turkey; 2 Department of Histology and Embryology, Koç University School of Medicine, Istanbul, Turkey

## Abstract

Harakiri (HRK) is a BH3-only protein of the Bcl-2 family and regulates apoptosis by interfering with anti-apoptotic Bcl-2 and Bcl-xL proteins. While its function is mainly characterized in the nervous system, its role in tumors is ill-defined with few studies demonstrating HRK silencing in tumors. In this study, we investigated the role of HRK in the most aggressive primary brain tumor, glioblastoma multiforme (GBM). We showed that HRK is differentially expressed among established GBM cell lines and that HRK overexpression can induce apoptosis in GBM cells at different levels. This phenotype can be blocked by forced expression of Bcl-2 and Bcl-xL, suggesting the functional interaction of Bcl-2/Bcl-xL and HRK in tumor cells. Moreover, HRK overexpression cooperates with tumor necrosis factor-related apoptosis-inducing ligand (TRAIL), a known tumor-specific pro-apoptotic agent. Besides, secondary agents that augment TRAIL response, such as the histone deacetylase inhibitor MS-275, significantly increases HRK expression. In addition, GBM cell response to TRAIL and MS-275 can be partly abolished by HRK silencing. Finally, we showed that HRK induction suppresses tumor growth in orthotopic GBM models in vivo, leading to increased survival. Taken together, our results suggest that HRK expression is associated with GBM cell apoptosis and increasing HRK activity in GBM tumors might offer new therapeutic approaches.

## Introduction

Glioblastoma multiforme (GBM) is the most common and aggressive brain tumor type and the median patient survival rate is approximately 15 months after diagnosis^1^. The term “Multiforme” describes one of the important GBM features, which is tumor heterogeneity affecting tumor cells’ morphologies, growth rates, and gene expression levels leading to variable responses of GBM cells to conventional therapies^1–3^.

In cancers, including GBMs, apoptotic programs are suppressed and tumor cells evade death through unique mechanisms. Deregulation of apoptosis disrupts the balance between cell proliferation and cell death, and thus leads to the development of cancer^4^. Accordingly, pro-apoptotic therapies triggering extrinsic pathway such, as TNF-related apoptosis-inducing ligand (TRAIL) or intrinsic pathway, such as BH3 mimetics bear the potential to eliminate cancer cells^5^.

Expression differences in the pro-apoptotic Bcl-2 members and the mitochondrial priming state of tumor cells is an important indicator of chemotherapeutic response^6,7^. Similarly, we have recently established TRAIL-sensitive and TRAIL-resistant subpopulations of tumors cells and observed marked expression differences between different Bcl-2 family members. Especially, BH3-only protein Harakiri (Hrk) gene was significantly upregulated in TRAIL-sensitive subpopulation of GBM cells. HRK is a sensitizer BH3-only protein and regulates apoptosis by interfering with anti-apoptotic Bcl-2 and Bcl-xL proteins and blocking their function^8^. Function of HRK is mainly described in the nervous system but its implications in tumorigenesis are not well studied^9–11^. Few studies show the suppressed expression levels of HRK in tumors by methylation^12,13^ and exogenous expression of HRK attenuates tumor growth in some cancers^12,14^. However, the functional role of HRK and its relation to other pro-apoptotic therapies like TRAIL has not been studied in GBM before. In this study, we investigated the effect of HRK on GBM cell apoptosis. We found that HRK is differentially expressed among established GBM cell lines. By employing gain-of- and loss-of-function approaches, we showed that HRK overexpression induces apoptosis in different GBM cells at different levels and attenuates tumor growth in vivo. Also, we showed that HRK-induced apoptosis could be inhibited by forced expression of Bcl-2 and Bcl-xL, suggesting the functional interaction of Bcl-2/Bcl-xL and HRK in tumor cells. Moreover, HRK overexpression cooperated with TRAIL in GBM cell lines using both intrinsic and extrinsic pathway for apoptosis. Lastly, we showed that HRK was one of the key players of the outcome of combinatorial therapies that involved TRAIL sensitization. Taken together, our results suggest that HRK is a key player in GBM cell death providing insight into the future design of pro-apoptotic therapies.

## Results

### HRK overexpression leads to cell death in GBM

**Fig. 1:**
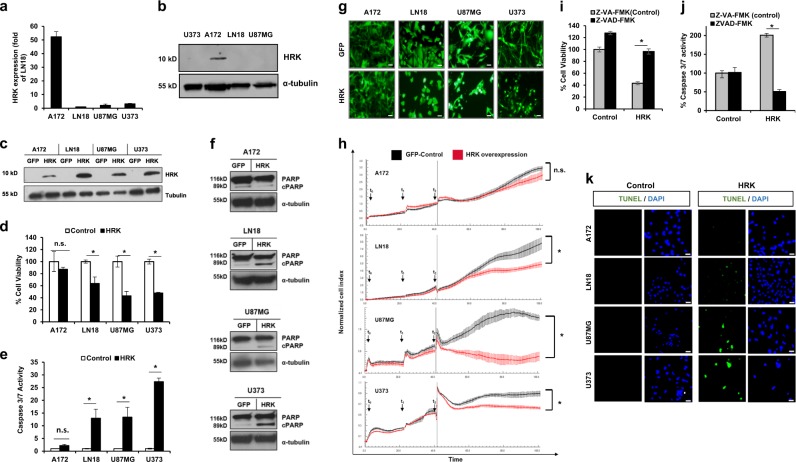
Harakiri overexpression leads to cell death. **a** Hrk is differentially expressed in four different established cell lines (A172, LN18, U87MG, U373). Values are normalized to the level of housekeeping gene, GAPDH. **b** Western blot analysis of endogenous HRK expression in A172, LN18, U87MG, U373 cell lines. **c** Western blot analysis of HRK in the whole cell lysate of GFP- and HRK-overexpressing GBM cell lines. **d**, **e** HRK overexpression decreases cell viability (**d**) and increases the activation of caspase 3/7 (**e**). (* denotes *p* < 0.05, n.s. denotes non-significant (*p* > 0.05), *t*-test). **f** Western blot analysis of PARP cleavage in GFP and HRK-overexpressing GBM cells. **g** Representative figures showing HRK-induced cell death in GBM cell lines, scale bars:100 µM. **h** Growth of GFP or HRK-expressing cells in long-term. t0 denotes the time of seeding, t1 denotes the time of viral transduction, t2 denotes the time of medium change. **i**, **j** General caspase inhibitor treatment (20 μM ZVAD-FmK or ZVA-FmK) recovers the HRK-induced changes in cell viability (**i**) and caspase activity (**j**). **k** TUNEL staining of GBM cell lines transduced with HRK or control vectors (green: TUNEL, blue: DAPI, scale bars: 10 µM)

As tumor cells’ apoptotic response might be correlated with the endogenous levels of apoptotic family members, we examined HRK expression levels in a panel of established GBM cell lines (A172, LN18, U87MG, and U373). Accordingly, A172 had the highest endogenous HRK expression compared to other cells lines, as measured by qRT-PCR (Fig. 1a) and western blot (Fig. 1b). Since the functional role of HRK has not been studied in GBMs and the endogenous expression of HRK was different among cell lines, we wished to test the role of HRK by overexpressing it in GBM cells. To this end, we generated a HRK overexpression vector and then infected the four established GBM cell lines with HRK and control GFP viruses. Western blot analysis validated the HRK overexpression compared to the GFP control (Fig. 1c). To test the functional effect of HRK expression on GBM cells, we first assessed cell viability and observed that HRK overexpression triggered cell death significantly in LN18, U87MG, and U373 but not in A172 cells as shown by cell viability assays and fluorescent images of cells displaying apoptotic morphologies (Fig. 1d, g). To assess whether Caspase activation was also involved in the observed reduction in cell viability, we measured the activity of effector caspases and demonstrated that HRK significantly increased caspase 3/7 activity in all GBM cell lines tested (Fig. 1e), however to different degrees. LN18, U87MG, and U373 cells had higher caspase activation compared to A172 cells in consistency with the viability results. Moreover, western blot analysis showed that HRK overexpression increased PARP cleavage significantly in LN18, U87MG, and U373 cells, but not in A172 cells (Fig. 1f). These results suggested that HRK plays a role in GBM cell apoptosis, and the downstream apoptotic events such as PARP cleavage is induced by HRK overexpression. Indeed, to test whether HRK-induced cell death was caspase-dependent, we employed general caspase inhibitor zVAD-FMK and observed that HRK-induced changes in cell viability (Fig. 1i) and caspase3/7 activity (Fig. 1j) were significantly reduced with zVAD-FMK treatment. To assess the long-term effects of HRK overexpression on GBM cell growth and proliferation, we performed a real-time cell growth analysis, where the GBM cells’ growth kinetics were observed for 72 h after viral transduction. Accordingly, HRK expression markedly reduced cell proliferation in long term; the kinetics of cell growth were different in LN18, U87MG, and U373 cells; and consistent with our previous experiments, where A172 cells responded the least to HRK expression (Fig. 1h). A further analysis with TUNEL staining also revealed HRK-induced apoptosis in GBM cell lines, particularly in U87MG and U373 cells, but not in A172 cells (Fig. 1k). Taken together, these results showed that HRK expression induces apoptosis in GBM cell lines to different degrees.

### Bcl-2 and Bcl-xL inhibits HRK-induced cell death in GBM

**Fig. 2:**
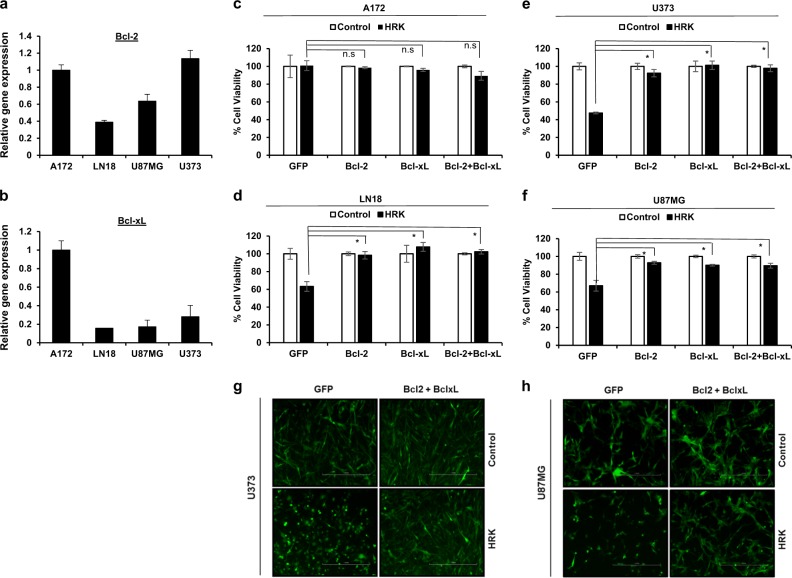
Bcl-2 and/or Bcl-xL overexpression inhibits HRK-induced death in GBM cells. **a**, **b** Gene expression levels of Bcl-2 and Bcl-xL in A172, LN18, U87MG, and U373 cells detected by qRT-PCR. Values are normalized to the level of housekeeping gene, GAPDH. **c**–**f** Cell viability effects  of HRK overexpression in GFP, Bcl-2 and/or Bcl-xL overexpressing A172 (**c**), LN18 (**d**), U373 (**e**) and U87MG (**f**) cells after 48 h HRK transduction. **g**, **h** Representative fluorescent images of U373 (**g**) and U87MG (**h**) cells transduced with HRK alone (left columns) or together with Bcl-2 and Bcl-xL (right columns) (scale bars:1000 µM) (* denotes *p* < 0.05, *t*-test). All experiments were performed in triplicates and representative of technical replicates has been shown

HRK is known as a sensitizer BH3 only protein and triggers apoptosis by neutralizing the anti-apoptotic Bcl-2 and Bcl-xL proteins^15^. However, the regulatory role of HRK in the apoptosis of cancer cells has not been studied before. To test the functional interaction between Bcl-2, Bcl-xL, and HRK in GBM cells, we first examined endogenous levels of Bcl-2 and Bcl-xL expression and observed that A172 cells expresses elevated levels of both Bcl-2 and Bcl-xL compared to LN18, U87MG, and U373 cells (Fig. 2a, b). We then generated GFP, Bcl-2 and/or Bcl-XL overexpressing GBM cells using bicistronic retroviral vectors encoding GFP. When we overexpressed HRK in Bcl-2 and/or Bcl-xL and GFP-expressing GBM cells, we observed that Bcl-2 and/or Bcl-xL overexpression inhibited HRK- induced apoptosis and led to the recovery of cell death in LN18 (Fig. 2d), U87MG (Fig. 2f, h), and U373 (Fig. 2e, g), but not in A172 cells (Fig. 2c). The overexpression of HRK in these cells did not affect the endogenous expression levels of Bcl-2 or Bcl-xL, as well as several other Bcl-2 family members, attesting that HRK overexpression does not indirectly regulate the levels of Bcl-2 family member expression (Supplementary Figure 1). These results suggest that HRK functions through regulating Bcl-2 and/or Bcl-XL activity in GBM cells.

### HRK overexpression decreases tumor growth in vivo

**Fig. 3:**
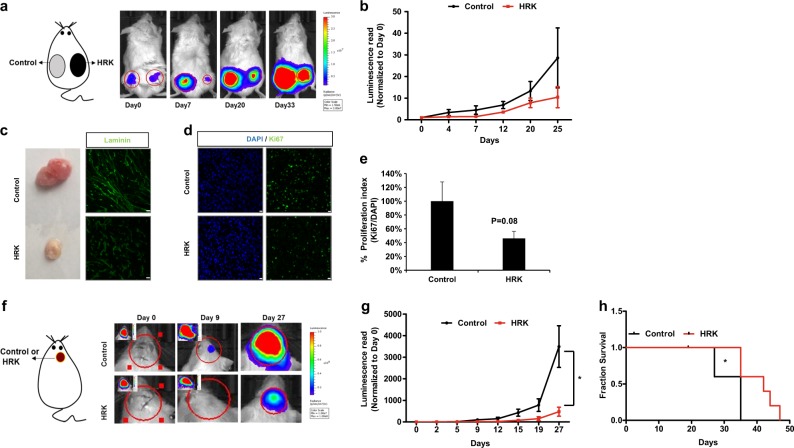
HRK overexpression decreases tumor growth in vivo. **a** Representative pictures of noninvasive bioluminescence imaging (BLI) of subcutaneous tumors over 33 days. U87MG cells transduced with Luciferase (Fluc)-mCherry (FmC) expressing vectors were post-infected with control or HRK vectors, and then injected subcutaneously into SCID mice (*n* = 5). **b** Quantification of tumor growth dynamics by BLI over time. **c**–**e**) Histological examination of tumors removed at the end of last imaging session. Laminin staining (green) to indicate vascularization (scale bars: 25 µM) (**c**), Ki67 (green) and DAPI (blue) staining to indicate proliferation and cell nuclei, respectively (scale bars:10 µM) (**d**), and quantification of proliferation by the number of Ki67-positive puncta over DAPI-positive puncta (n = 5) (**e**). **f**) Representative pictures of BLI of intracranial tumor growth over 27 days. U87MG-FmC cells post-transduced with control or HRK vectors were injected intracranially into SCID mice (n = 5 each group) and tumor growth dynamics was measured over time. **g**) Quantification of tumor growth dynamics by BLI over time. **h**) Kaplan-Meier survival graphs of intracranial tumor bearing mice implanted with Control- or HRK-expressing cells. (* denotes p < 0.05, ANOVA)

To examine the HRK expression effect on tumor growth in vivo, we first generated firefly luciferase (Fluc) and mCherry expressing U87MG GBM cells by transducing GBM cells with Fluc-mCherry expressing viral vectors (FMC). FMC expressing U87MG cells showed mCherry protein under fluorescence microscope and displayed Fluc activity (Supplementary Figure 2). Subcutaneous injection of the cells in immunocompromised mice and long-term analysis of tumor growth revealed that HRK expression attenuates tumor growth in vivo (Fig. 3a, b). In the tumor tissues, immunofluorescence staining showed that HRK-expressing tumors were smaller in volume, had less vascularity as shown by Laminin staining (Fig. 3c), and less Ki-67 proliferation index compared to control tumors (Fig. 3d, e). Effect of the HRK overexpression on tumor growth was also tested in orthotropic in vivo model of GBM, where U87MG-FMC cells were intracranially injected (Fig. 3f). Accordingly, HRK overexpressing tumors displayed slower growth (Fig. 3f, g), and mice bearing HRK-expressing tumors survived significantly longer than those with control tumors [median survival of 42 (HRK) vs 35 (Control) days] as displayed by Kaplan-Meier curves (Fig. 3h). Together, these results suggest that GBM tumors with high HRK expression have less growth ability.

### HRK overexpression cooperates with TRAIL in GBM cells

**Fig. 4:**
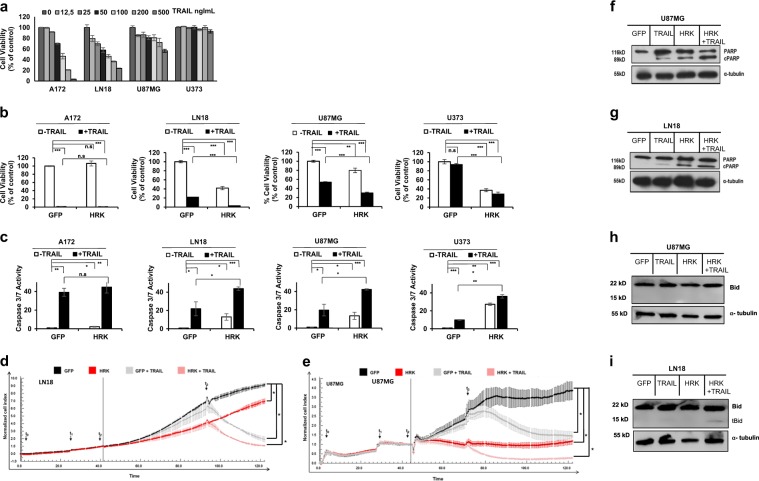
HRK overexpression cooperates with TRAIL to induce cell death in GBM cells. **a** GBM cells have differential response to TRAIL as measured by cell viability assays of cells in response to TRAIL treatment (0-500 ng/ml) for 24 h. **b** Cell viability analysis of GFP- or HRK- overexpressing A172, LN18, U87MG, and U373 cells upon 24 h TRAIL treatment (50 ng/ml). **c** Caspase 3/7 activity analysis of GFP- or HRK- overexpressing A172, LN18, U87MG, U373 GBM cells after 3 h TRAIL treatment (TRAIL concentrations were 20 ng/ml, 20 ng/ml, 50 ng/ml, 200 ng/ml for each cell line respectively). (*, **, *** denote *p* < 0.05, *p* < 0.01, *p* < 0.001, *t*-test) **d**, **e** Effect of HRK and TRAIL cooperation on the growth of U87MG cells in long-term. GBM cells were seeded (t0) and 24 h later were transduced with HRK or GFP viruses (t1). After 24 h, media was refreshed (t2). After 40 h, TRAIL was added (t3) as 25 ng/ml for LN18 (**d**) and 50 ng/ml TRAIL for U87MG (**e**) cells (* denotes *p* < 0.05, ANOVA). **f**–**i** Western blot analysis of cleaved PARP and Bid protein upon HRK induction and TRAIL treatment (50 ng/ml) in LN18 and U87MG cells. All experiments were performed in triplicates and representative of technical replicates has been shown

TRAIL is a ligand that induces extrinsic apoptosis specifically in cancer cells^16^. Although TRAIL selectively kills cancer cells, the response to TRAIL is very different among different cancer cell lines. To further examine the effect of HRK expression in relation to TRAIL response of GBM cells, we first characterized the TRAIL response of our established GBM cell lines (A172, LN18, U87MG, and U373). Accordingly, cells were categorized according to their TRAIL response as sensitive (A172), mid-sensitive (U87MG and LN18) and resistant (U373) (Fig. 4a). To examine the combinational effect of HRK overexpression and TRAIL in GBM cells, we treated control (GFP-expressing) or HRK-expressing GBM cells with TRAIL. Cell viability analysis showed that while TRAIL reduced the viability of TRAIL-sensitive A172 cells, HRK did not cause an additional death, consistent with previous results. In TRAIL-resistant U373 cells, viability was significantly decreased by HRK overexpression, but it was not affected by TRAIL treatment. In contrast, HRK overexpression cooperated with TRAIL in U87MG and LN18 TRAIL mid-sensitive GBM cell lines, where HRK-overexpressing cells had better response to TRAIL treatment (Fig. 4b). For further examination, we measured TRAIL-induced caspase 3/7 activation in HRK-overexpressing GBM cells. Caspase 3/7 activity measurements and cell viability analysis gave similar results and showed that HRK and TRAIL collaborated to induce apoptosis in U87MG and LN18 GBM cell lines, but not in A172 and U373 cells (Fig. 4c). To determine the long-term effect of TRAIL and HRK cooperation in LN18 and U87MG cells, we employed real-time cell growth assays and acutely transduced GBM cells with GFP or HRK encoding viruses (time designated as t1) followed by medium change (t2) and TRAIL treatment (t3). As seen in the plots, TRAIL treatment decreased the number of measurable live cells in this system, and TRAIL and HRK together led to reduced cell number in both LN18 (Fig. 4d) and U87MG (Fig. 4e) cells. As a marker of apoptosis, we examined PARP cleavage in GFP- or HRK-expressing LN18 and U87MG cells and showed that HRK overexpression with TRAIL treatment increased the cleaved PARP compared to the HRK overexpression or TRAIL treatment alone (Fig. 4f, g). To examine the connection between extrinsic and intrinsic apoptosis activation, we examined Bid cleavage and observed that HRK and TRAIL cooperate to induce Bid cleavage levels in LN18 cells, but not U87MG cells (Fig. 4h, i). Together these results showed that TRAIL and HRK cooperate to cause increased cell death.

### HRK is partly responsible for apoptotic sensitization

**Fig. 5:**
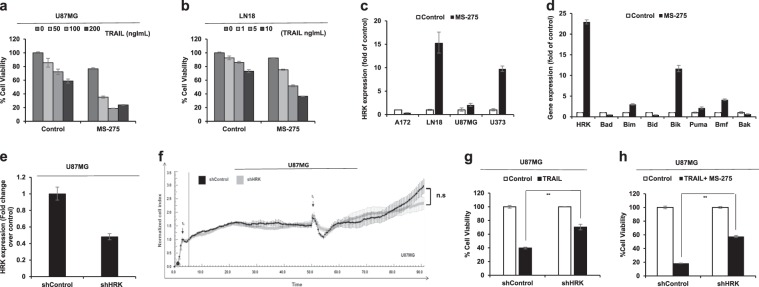
HRK expression is partly required for TRAIL response and TRAIL sensitization by secondary agents. **a**, **b** Viability analysis of U87MG cells (**a**) and LN18 cells (**b**) upon differential doses of TRAIL and MS-275 (5 μM) combination. **c** qRT-PCR analysis of Hrk expression in four established GBM cell lines (A172, LN18, U87MG, U373) upon MS-275 treatment (5 μM). **d** qRT-PCR analysis of Bcl-2 family member genes when treated with MS-275 (5 μM). **e** qRT-PCR analysis of Hrk expression in shControl and shHRK transduced U87MG cells. Values are normalized to the level of housekeeping gene, GAPDH. **f** Long-term cell growth analysis of shControl and shHRK U87MG cells (t0: time of cell seeding, t1: time of media change). **g** Cell viability analysis of shControl and shHRK transduced U87MG cells upon TRAIL treatment (75 ng/ml). **h** Cell viability analysis of shControl and shHRK transduced U87MG cells upon TRAIL (75 ng/ml) and MS-275 (5 μM) combination. (*, **, *** denote *p* < 0.05, *p* < 0.01, *p* < 0.001, *t*-test). All experiments were performed in triplicates and representative of technical replicates has been shown

Even though TRAIL response is differential among GBM cell lines, many groups previously showed that TRAIL sensitization is possible^17^. A histone deacetylase inhibitor, MS-275, which is known to increase DR4 and DR5 expression, sensitizes TRAIL-resistant GBMs to TRAIL^18^. However, the exact mechanism behind MS-275 mediated sensitization prompts further investigation. We first examined the TRAIL sensitization effect of MS-275 in U87MG and LN18 TRAIL mid-sensitive GBM cell lines upon differential doses of TRAIL treatment. Cell viability assay showed that MS-275 sensitized U87MG cells (Fig. 5a) and LN18 cells (Fig. 5b) to TRAIL. Since HRK cooperates with TRAIL, it may contribute to TRAIL sensitization mechanism of MS-275. To this end, we first examined the HRK expression changes upon MS-275 treatment. qRT-PCR analysis demonstrated that Hrk expression markedly increases with MS-275 treatment in most GBM cell lines (LN18, U87MG, and U373), but not in A172 (Fig. 5c). Interestingly, when we examined the effect of MS-275 on other related Bcl-2 family members (Bad, Bim, Bid, Bik, Puma, Bmf, and Bak), we observed major upregulations of some of these genes in response to MS-275 treatment. However, the level of Hrk upregulation was the highest among these genes (Hrk upregulation was more than 22 fold, compared to 2.9 fold, 11.6 fold, 2.1 fold and 4.1 fold for Bim, Bik, Puma, and Bmf genes, respectively) (Fig. 5d). To then elucidate the requirement of HRK for TRAIL-induced apoptosis, we undertook a loss-of-function approach and generated a silencing shHRK vector in a lenti-viral backbone to knock down the expression of Hrk. qRT-PCR analysis demonstrated that shHRK reduced Hrk mRNA expression to ~50% compared to shControl vector (Fig. 5e). Upon selection of stable cell lines with HRK silencing, we performed long term cell growth analysis to assess the effect of HRK knockdown alone on the proliferative capacity of the cells and found that shHRK did not affect cell growth (Fig. 5f). Then, to investigate the requirement of HRK in TRAIL-induced apoptosis, we performed cell viability analysis in shHRK and shControl transduced U87MG cells upon TRAIL treatment. Analysis showed that HRK knockdown partially prevents TRAIL-induced apoptosis in U87MG cells (Fig. 5g). These results suggest that HRK is partly involved in TRAIL-induced apoptosis of GBM cells. We also performed similar experiments in the highest HRK-expressing A172 cells but shHRK mediated silencing was not sufficient in these cells (data not shown). Therefore we employed CRISPR/Cas9-based gene silencing and observed that TRAIL sensitivity was markedly reduced when HRK expression was reduced in A172 cells with multiple gRNAs (Supplementary Figure 3). We then tested the requirement of HRK for TRAIL sensitization effect of MS-275 upon combined treatment of TRAIL and MS-275 (Fig. 5h). Accordingly, we showed that silencing of HRK in GBM cells reduced MS-275 mediated TRAIL sensitization. These results suggest that HRK is one of the key factors responsible for TRAIL response and sensitization.

### HRK is differentially expressed in different GBM cell subpopulations and cooperates with TRAIL in primary GBM cell lines

**Fig. 6:**
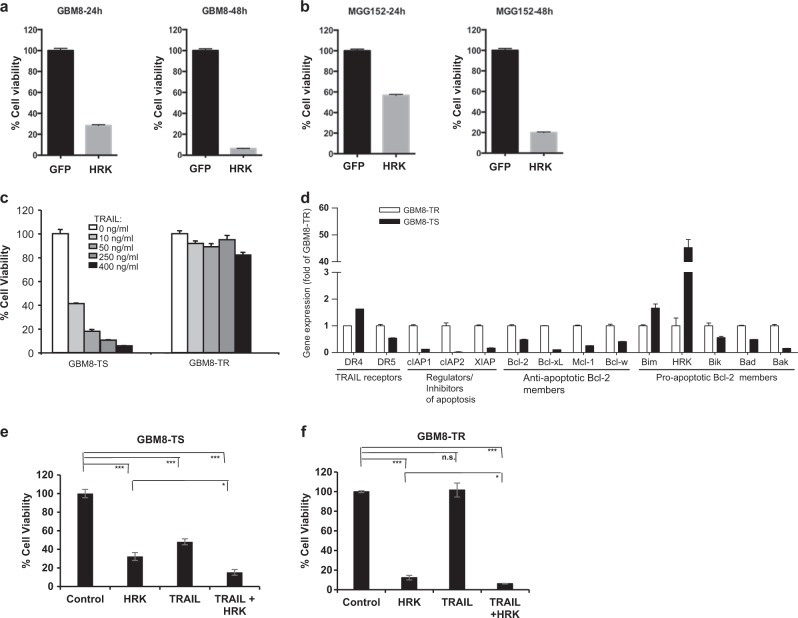
HRK is differentially expressed in different GBM cell subpopulations and cooperates with TRAIL in primary GBM cell lines. **a**, **b** HRK overexpression decreases the viability of primary GBM cell lines, GBM8 (**a**) and MGG152 (**b**), in a time-dependent manner. **c** TRAIL response of an isogenic GBM cell line (GBM8) pair selected for TS (TRAIL sensitive) and TR (TRAIL resistance) subpopulations that were exposed with differential doses of TRAIL (0, 10, 50, 250, 400 ng/mL) were assessed. **d** qRT-PCR analysis of apoptotic members in GBM8-TS and GBM8-TR subpopulations was performed. **e**, **f** HRK overexpression decreases cell viability and cooperates with TRAIL in TRAIL-resistant (**e**) and TRAIL-sensitive (**f**) subpopulation of a primary GBM cells. (n.s., *, *** denote non-significant, *p* < 0.05, *p* < 0.001, respectively, *t*-test)

To expand our findings on HRK to primary GBM cell lines, we employed two different cell line models. HRK overexpression led to marked decrease in the viability of both GBM8 and MGG152 cell lines in a time-dependent manner (Fig. 6a, b). Among these, isogenic subpopulations of GBM8 cells that were generated under different culture conditions were available^19^, where they exhibited markedly different responses to TRAIL (Fig. 6c), as a testament to GBM cell heterogeneity. In these cells, we observed that anti-apoptotic Bcl-2 family members and inhibitors were downregulated and pro-apoptotic members and TRAIL receptors were upregulated in TRAIL-sensitive subpopulation compared to the TRAIL-resistant subpopulation. Notably,  Hrk gene expression was significantly higher in TRAIL-sensitive subpopulation of GBM8 cells (>40 fold of TRAIL-resistant subpopulation) (Fig. 6d). We also showed that HRK overexpression in these cells led to their death alone and in combination with TRAIL, suggesting that HRK can induce apoptosis in TRAIL-resistant and -sensitive subpopulations of GBM cells (Fig. 6e, f).

## Discussion

In this study, we examined the role of a pro-apoptotic BH3-only Bcl-2 family member protein, Harakiri (HRK) in GBM cell apoptosis. We have shown that HRK expression induces cell death in GBM cells in vivo and in vitro, and that its function can be inhibited by Bcl-2 and/or Bcl-xL expression in GBM cells. In addition, HRK can cooperate with extrinsic apoptosis-inducing ligand TRAIL in a subset of GBM cell lines and HRK silencing partially prevents TRAIL-induced apoptosis. Besides, HRK silencing can abrogate the TRAIL-sensitization ability of secondary agents such as MS-275. Taken together, these results suggest a novel role for HRK as a key regulator of apoptosis and apoptotic sensitization in GBM cells.

In the literature, there have been a few studies examining the role of HRK expression in tumors, and these focused on prostate, breast, ovarian cancers, and melanoma^12,14^. There are few studies that implicated HRK indirectly in GBM survival, however these studies did not provide direct and in-depth interrogation of functional role of HRK in GBMs^20,21^. To our knowledge, ours is the first study to address the functional role of HRK in GBM thoroughly. Therefore, our findings have the potential to open new avenues for the potential use of pro-apoptotic therapies in GBMs in the future. HRK is a sensitizer BH3-only protein and neutralizes Bcl-2 and Bcl-xL to trigger cell death^8,22^. In our study, we validated the functional interaction between HRK and Bcl-2/Bcl-xL in GBM cells as HRK-induced cell death was blocked when Bcl-2 and/or Bcl-xL were introduced.

This study also examined the relation between HRK expression and extrinsic apoptosis induction by TRAIL. Using various established GBM cell lines with differential TRAIL response, as well as isogenic subpopulations of a primary GBM cell line with different TRAIL response thresholds, we showed that HRK endogenous expression is very high in TRAIL-sensitive cells. However, since the balance between anti-apoptotic and pro-apoptotic proteins determines the outcome of the death signal, the expression levels of Bcl-2 family members indicate how close cells are to apoptotic threshold. Indeed, we showed that the GBM cell line that expressed the highest level of HRK (A172) had the highest Bcl-2 and Bcl-XL expression^7^, however further analyses on the levels of all Bcl-2 family gene expression as well as their functionality are required. Recently, a new technique called BH3 profiling is used to determine the mitochondrial priming of cancer cell lines, where tumor cells are exposed to a specific BH3 peptide and mitochondrial outer membrane permeabilization is detected^6^. To this end, employing this strategy to determine the mitochondrial priming state of our GBM cells in relation to their response to TRAIL and HRK expression will be of great interest to dissect out the HRK-induced changes in GBM cells in the future.

In the literature, Hrk expression is reported to be repressed by loss of heterozygosity and promoter hypermethylation in some cancers including GBM^12,21,23^. Thus, endogenous Hrk expression differences among GBM cell lines might be due to the differences in methylation status of the *hrk* gene and opening  Hrk expression via epigenetic modulation in the repressed *hrk* gene loci will be an interesting avenue to pursue in the future. To this end, we have evidence that Hrk is under epigenetic control in the GBM cell lines we used, as we observed marked  Hrk upregulation in response the DNMT1 inhibitor, 5-azacytidine in all cell lines (Supplementary Figure 4).

We used a gain-of-function approach to examine the functional outcome of HRK induction in our study. In TRAIL-sensitive A172 GBM cell line with the highest endogenous HRK levels, exogenous HRK expression failed to cause cell death, however in all other cell lines HRK expression led to apoptosis. This could be because expression of exogenous HRK expression might not be enough to change the apoptotic threshold in A172 mitochondria. Alternatively, A172 cells can be Type I cells, which use extrinsic pathway for apoptosis and mitochondrial pathway might not be involved. For example, while Bcl-2/Bcl-xL overexpression completely inhibits TRAIL effects in U87MG cells, it fails to show complete recovery in A172 cells (Supplementary Figure 5) again supporting this notion. However, further studies are needed to assess these possibilities. In-TRAIL resistant U373 GBM cell line, we showed that HRK overexpression alone induces cell death. This result suggests that U373 cells are close to the mitochondrial apoptotic threshold and exogenous HRK expression is sufficient to trigger the activation of mitochondrial apoptosis pathway. In addition, this suggests that U373 cells can give better response to chemotherapeutic agents, which usually activate mitochondrial apoptosis pathway. Such agents include Bcl-2/Bcl-XL inhibitors^24^, and other BH3 mimetics^25^. In parallel, we showed that ectopic HRK expression can trigger cell death also in TRAIL mid-sensitive LN18 and U87MG cells and cooperate with TRAIL treatment. In these cells, knockdown of HRK partly prevents TRAIL-induced apoptosis, suggesting that extrinsic and intrinsic arms of apoptosis are in interplay. Since LN18 and U87MG cells are both responsive to HRK overexpression and TRAIL treatment, they might be categorized under Type II cells and the apoptotic mechanisms regulated by HRK in such tumor cell types might be different than that of A172 cell like Type I cells. We also have preliminary evidence that HRK as a regulator of intrinsic apoptosis can also cooperate with other extrinsic ligands such as FasL (Supplementary Figure 6), and the mechanistic details of such cooperation prompts further studies.

Innate or acquired TRAIL resistance is a major problem in TRAIL-based therapies and combinatorial approaches that aim to overcome such resistance is a promising therapeutic approach. One of the well-known TRAIL-sensitizing secondary agents is MS-275, a histone deacetylase inhibitor^18^. While the sensitization mechanism of MS-275 and similar agents involve the upregulation of death receptors and/or downregulation of anti-apoptotic proteins, exact mechanisms are still ill-defined. In this study, we have shown that HRK expression is responsive to MS-275 treatment and that the knockdown of HRK inhibits the TRAIL sensitizing effect of MS-275. These results suggest that HRK can be key factor for the sensitization mechanism of MS-275.

Finally, we showed that HRK expression led to slower tumor growth in subcutaneous and orthotopic tumor models in vivo. Accordingly, HRK-expressing tumors had less proliferative capacity, less vascularization and mice implanted with HRK-expressing tumors lived significantly longer. Designing and using regulatable systems where the timing of HRK induction is controlled in vivo, will be of great future interest to examine how established tumors react to induced HRK expression. To our knowledge, ours is the first study to show the effect of HRK expression in GBM models, which suggest that induction of HRK expression either by secondary agents or by targeted delivery can be promising future approaches. Although HRK expression has not been associated as a prognostic factor in GBMs in earlier studies^26^, forced induction of HRK in tumors can have possible clinical applications. For example, GBMs can be treated with HRK protein^27^ or *hrk* cDNA can be delivered in nanoparticles^28^ to induce cell death in GBMs.

Taken together, our findings provide a novel mechanism of apoptosis regulation in GBM cells, that is by HRK and its regulation. Therefore, further understanding of the biology of HRK and finding new drugs based on HRK expression and function might open up new windows in designing effective GBM therapies.

## Materials and methods

### Cell Culture and reagents

A172, LN18, U87MG, and U373 GBM cell lines and human embryonic kidney 293 T cells were obtained from American Tissue Type Culture Collection (USA) and cultured in DMEM medium (Gibco, USA) with %10 fetal bovine serum (Gibco, USA) and %1 penicillin-streptomycin (Gibco, USA). MGG152, GBM8-TR, and GBM8-TS cells were kindly gifted by Dr. Hiroaki Wakimoto (Massachusetts General Hospital, Boston, MA)^19^. GBM8-TR cells were cultured in DMEM (Gibco) supplemented with 10% FBS and 1% penicillin/streptomycin. GBM8-TS cells were cultured in neurobasal medium (Gibco) supplemented with 3 mmol/L of L-Glutamine (Mediatech), B27 (Invitrogen/GIBCO), 2 μg/mL of heparin (StemCell Technologies), 20 ng/mL of human EGF (R&D Systems), and 20 ng/mL of human FGF-2 (fibroblast growth factor; PeproTech). All cells were grown in 37 °C, 5% CO_2_ in a humidified incubator. TRAIL was commercially supplied (Enzo Life Sciences, US) or produced from 293T cells as described^29^. Stock of MS-275 (Cayman Chemicals, USA) was prepared in DMSO. ZVAD-FmK and ZVA-FMK were prepared in DMSO (Promega, USA).

### Cloning and packaging of HRK overexpression and silencing vectors

pcDNA3.1 HRK vector was kindly provided by Marta Miaczynska (International Institute of Molecular and Cell Biology, Poland). *hrk* cDNA sequence of the vector was cloned into lenti-viral backbone pLenti-PGK-puro (w529-2) (Addgene plasmid #191068) by using Gateway cloning^30^. shRNA sequences targeting first exon of *hrk* gene were designed using RNAiCodex program^31^. These oligos were PCR-amplified by using following primers having compatible restriction ends with backbone vector, pSMP. Forward: 5’GATGGCTGCTCGAGAAGGTATATTGCTGTTGACAGTGAGCG-3’, Reverse: 5’-CCCTTGAACCTCCTCGTTCGACC-3’. PCR products were cloned into pSMP retro-viral backbone as described^32^. All vectors were verified by sequencing. All vectors were packaged and lentiviral or retroviral particles were generated as described^18,32^.

### Cell viability assay

All cell viability assays were performed with ATP-based Cell Titer-Glo® (CTG) Luminescent Cell Viability Assay (Promega, USA) according to the manufacturer’s instructions and as described^33^. To determine the effects of HRK overexpression on GBM cell viability, cells were seeded into 96 well black bottom plate as 10.000 cells/per well in triplicates and infected with HRK or GFP viruses. After 36 h post-transduction, cell viability was measured by CTG. In order to examine the combination effect of HRK and TRAIL treatment, after 36 h post-transduction, cells were treated with TRAIL (20-200 ng/ml) and cell viability was measured after 24-hour incubation.

### Caspase 3/7 activity assay

Cells were seeded into 96 well black bottom plate as 10.000/per well in triplicates and infected with HRK. 36 h post-transduction, caspase activity was measured by Caspase 3/7 Glo Assay (Promega, USA) according to manufacturer’s instructions. In order to examine the combination effect of HRK and TRAIL treatment, 36 h post-transduction, cells were treated with TRAIL (20-200 ng/ml) for 3 h and Caspase 3//7 activity was determined.

### Quantitative RT-PCR

RNA isolation and cDNA synthesis were performed as described^33^. Relative Hrk gene expression levels were detected using LightCycler® 480 SYBR Green I Master (Roche, Germany). Following primer pairs were used: GAPDH: Forward: 5’-AGCCACATCGCTCAGACAC-3’, Reverse:5’-GCCAATACGACCAAATCC-3’. Hrk: Forward: 5’-AGGTTGGTGAAAACCCTGTG-3’, Reverse:5’-GCATTGGGGTGTCTGTTTCT-3’. All other primers are indicated in Supplementary Table 1.

### Western blotting

Following infection of HRK and GFP viruses and also TRAIL treatment, GBM cells were lysed using NP40 lysis buffer supplemented with 0.5 mM PMSF, 1X phosphatase inhibitor cocktail (PhoSTOP, Roche, Switzerland) and 1X protease inhibitor cocktail (cOmplete Protease Inhibitor Cocktail Tablets, Roche, Germany). Protein quantification was performed with BCA Protein Assay kit (Life Technologies,USA). For immunoblotting, 20-25 μg proteins were separated on SDS-PAGE gel and transferred to the PVDF membrane Trans-Blot® Turbo™ RTA Mini PVDF Transfer Kit (#170-4272, Biorad, USA). Membrane was immunoblotted with antibodies against α-Tubulin (T9026, Sigma-Aldrich), HRK (sc-6972, Santa Cruz Biotech), PARP (9542, Cell Signaling), and Bid (2002, Cell Signaling) and detected by secondary antibodies conjugated to HRP as described^33^.

### Real-time cell growth analysis

xCELLigence RTCA Station and analyzer (ACEA Bioscience, USA) were used for the long-term growth analysis. 2500 cell/well were seeded into E-plate 96 at (t0) and transduced with GFP and HRK viruses at t1. Viruses were removed at t2. Cell viability was measured with 30-minute intervals for 140 h. To assess the combination effect of TRAIL and HRK, HRK infected U87MG and LN18 cells were treated with TRAIL (25-50 ng/ml) at t3 and impedance of each well was measured with 30 minute intervals for 140 h. The data was analyzed using xCELLigence real-time cell analyzer.

### TUNEL staining

GBM cells were seeded to 12 well plates (30.000 cell/well) on glass coverslips. 24 h after infection with control or HRK-encoding lentiviruses, plates were washed 3 times with PBS. Air dried cells were fixed by 300 ul fixation solution (4% PFI in PBS, pH 7.4, freshly prepared) at 4 °C for 1 h. After rinsing with PBS followed by incubation in 300  μl Blocking solution (3% H_2_O_2_ in methanol) for 10 min at RT, permeabilization was performed (0.1% TritonX-100 in 0.1% sodium citrate, freshly prepared) at RT. Area around sample was dried and 50 μl  TUNEL reaction mixture (5 μl enzyme solution mixed with 45 μl label solution) (11684817910, Roche, US) was added for incubation for 60 min in 37 °C. After mounting in DAPI-Vectashield medium, slides were sealed and visualized by fluorescence microscopy.

### In vivo experiments

In this project, SCID mice housed and cared in appropriate conditions of Koç University Animal Facility were used and all protocols were approved by the institution boards of Koç University (HADYEK#2014-22). Firefly Luciferase (Fluc) and mCherry expressing stable U87MG cells were generated by viral transduction as described^18^. mCherry expression was validated under the fluorescence microscopy and Fluc activity was validated by utilizing in vitro luminescence assay and Synergy Biotek Plate reader. Before implantation, Fluc-mCherry expressing U87MG cells were transduced with pLenti-PGK-HRK-puro or control viruses. For subcutaneous tumor implantation, 2 × 10^6^ HRK-expressing or control U87MG cells were injected in 100 μl PBS per mouse (*n* = 5/group). For orthotopic model, SCID mice were implanted with 1 × 10^5^ HRK-expressing or control U87MG cells in 7 μl PBS intracranially as described^34^. Progression of tumors was monitored up to 40 days by repeated noninvasive bioluminescence imaging (IVIS Lumina III). Accordingly, mice were injected with 150 μg/g body weight of D-Luciferin intraperitoneally and sum of the photon counts of tumor regions were obtained. At the end, the tumors were dissected and analyzed with immunohistochemistry.

### Histological analysis

Samples were fixed by 4% paraformaldehyde for 24 h followed by 20% and 30% (wt/vol) sucrose treatment for cryosectioning. Consecutive cryosections (10 μm) were used for hematoxylin/eosin staining and fluorescent stainings. Laminin (ab11575, Abcam, US) followed by fluorescent conjugated secondary antibody of Alexa fluor 488 GAM (Cell Signaling, US) was used for evaluation of vascular structures. Ki-67 was used to assess proliferating cells in the tissues (Cell Signaling, US). DAPI (1 μg/ml) was used in mounting medium. Images were taken under a Nikon Eclipse 90i confocal microscope and a Zeiss axioscope.

### Statistical analysis

Student *t*-test was used for analysis of data while comparing two groups. Data were plotted as mean  ± SEM and differences were considered significant at *p* < 0, 05. ANOVA was used to calculate significance of real time cell growth Xcelligence experiments and in vivo tumor growth experiments. Overall survival of mice was analyzed by Kaplan–Maier survival analysis.

## Supplementary information

Supplementary Information

Supplementary Figures
