# Supplementary material for: The pro-apoptotic Bcl-2 family member Harakiri (HRK) induces cell death in glioblastoma multiforme
Source: Cell Death Discov. 2019 Feb 8;5:64. doi: 10.1038/s41420-019-0144-z (PMC6368544; doi:10.1038/s41420-019-0144-z)
Supplement: Supplementary file 1 — Supplementary Information [file 41420_2019_144_MOESM1_ESM.docx]

**SUPPLEMENTARY INFORMATION**

**CRISPR/Cas9 experiments**

A172 cells were transduced with PlentiCas9 virus to get stable Cas9 expression. Cells were selected with Blasticidin for 7 days. Stably Cas9 expressing A172 cells were transduced with pLentiGuide HRK gRNA#1, HRK gRNA#2 and HRK gRNA#3 (sequences indicated below) viruses. As a negative control, GFP targeting T1 and T2 gRNAs were used ^1^. gRNA transduced cells were kept in culture for 18 days, until maximum Cas9 endonuclease activity was obtained. After Cas9 activity, cells were counted and seeded as 10000 cells/well in 96 well plates. A day after, cells were treated with different concentrations of TRAIL and cell viability was measured using CTG assay 24 hours after TRAIL treatment.

| **gHRK1_1** | CACCGCGTCGCCTAGCGCCTTGAGC | | | |
| --- | --- | --- | --- | --- |
| **gHRK1_2** | AAACGCTCAAGGCGCTAGGCGACGC | | | |
|  |  |  |  |  |
| **gHRK2_1** | CACCGAGCTGCACCAGCGCACCATG | | | |
| **gHRK2_2** | AAACCATGGTGCGCTGGTGCAGCTC | | | |
|  |  |  |  |  |
| **gHRK3_1** | CACCGTTGGCTGTGCGCGGCCGCGC | | | |
| **gHRK3_2** | AAACGCGCGGCCGCGCACAGCCAAC | | | |

**FasL viability assay**

Fas Ligand was purchased from Sigma (Cat. No. SRP3036). Cells that were previously transduced with control or HRK viruses were seeded to 96 well plates and the following day treated with FasL for 72 hours. Cell viability was detected by Cell Titer Glo Assays.

**5-Azacytidine treatment**

All cells were treated with increasing doses of 5-Azacytidine (Selleckchem) for 24 hours and expression levels of HRK were measured as described in the manuscript. Accordingly, RNA was extracted, cDNA was synthesized and qRT-PCR was performed with HRK specific primers. GAPDH was used for normalization.

**SUPPLEMENTARY FIGURE LEGENDS**

**Supplementary Figure 1:** **Expression of Bcl-2 family genes in HRK overexpressing cell lines**. Values are normalized to the level of housekeeping gene, GAPDH (* denotes p<0.001, n.s. denotes non-significant (p>0.05), ANOVA). All experiments were performed in triplicates and representative technical replicates were shown.

**Supplementary Figure 2:** **Generation of Firefly luciferase (Fluc)-mCherry (FmC) expressing U87MG cells**. Luminescence signal of increasing number (0-25.000) of FmC expressing U87MG cells were measured by luminescence assay upon 100 ng/ml luciferin treatment. Synergy Biotek Plate Reader was used.

**Supplementary Figure 3: a)** Western Blot of A172 HRK knockout cells. T1 and T2 are GFP-targeting gRNAs. **b)** Cell viability results of different dosages of TRAIL treatment of 24 hours.

**Supplementary Figure 4:** Cells were treated with increasing doses of 5-Azacytidine for 24 hours and expression levels of HRK were measured. GAPDH was used for normalization.

**Supplementary Figure 5:** **Bcl-2 and Bcl-xL overexpression inhibits TRAIL-induced apoptosis in GBM cell lines**. Viability analysis of TRAIL treatment (24 hour) in GFP, Bcl-2 and Bcl-xL overexpressing A172 (a) and U87MG (b) cell lines.

**Supplementary Figure 6:** **a)** Cell viability of different dosages of FasL on A172, U373, U87MG and LN18 cells. **b)** Combination treatment of FasL (50 ng/ml) and HRK on LN18 and U87MG cells.

**Supplementary Table 1.** Primer sequences used in qRT-PCR experiments.

**References:**

1. Mali, P. *et al.* RNA-guided human genome engineering via Cas9. *Science* **339,** 823–6 (2013).
