# Supplementary material for: The pro-apoptotic Bcl-2 family member Harakiri (HRK) induces cell death in glioblastoma multiforme
Source: Cell Death Discov. 2019 Feb 8;5:64. doi: 10.1038/s41420-019-0144-z (PMC6368544; doi:10.1038/s41420-019-0144-z)
Supplement: Supplementary file 2 — Supplementary Figures [file 41420_2019_144_MOESM2_ESM.pptx]

## Slide 1
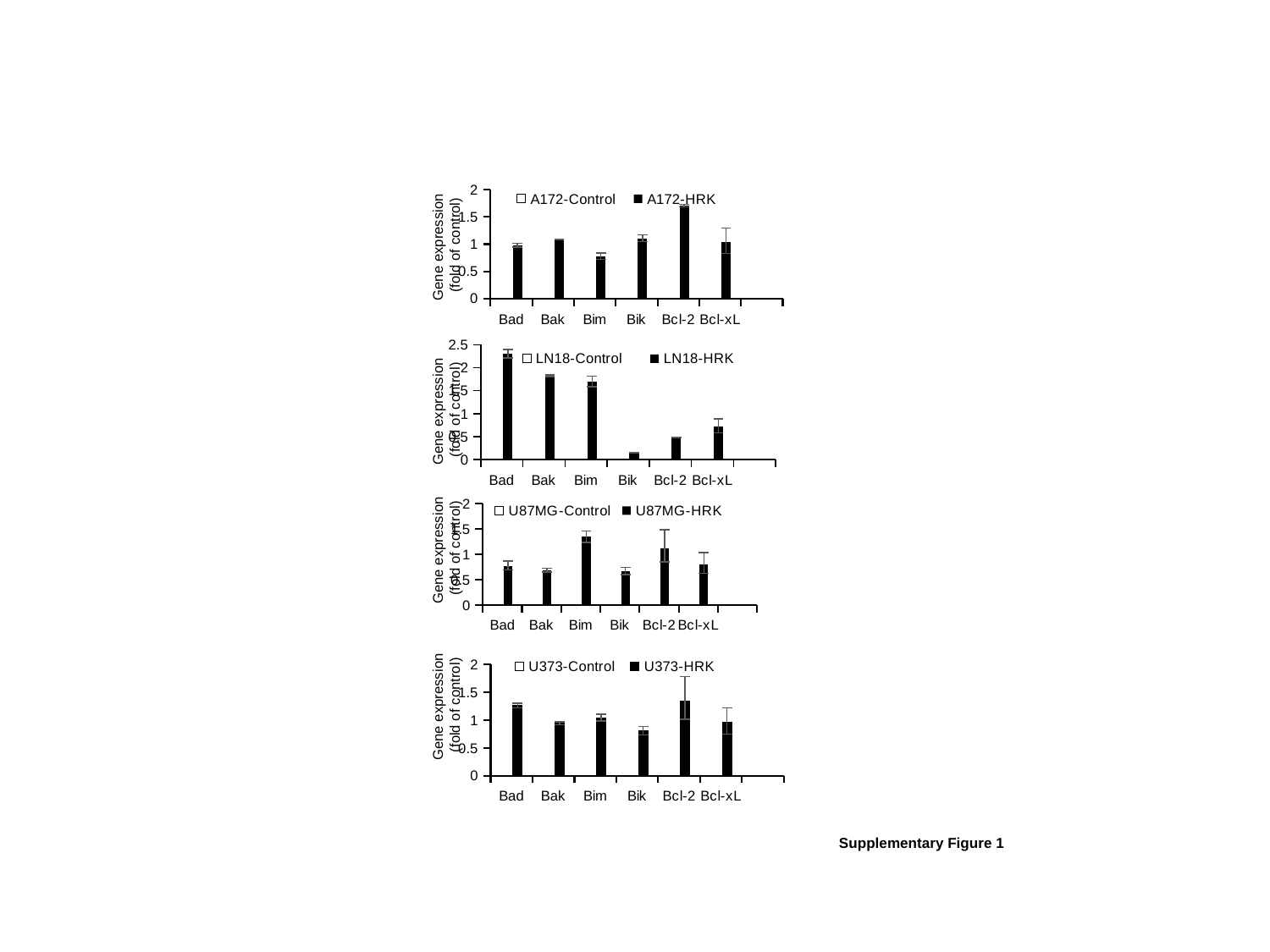

### Chart
| Category | A172-Control | A172-HRK |
|---|---|---|
| Bad | 1.0 | 0.979420297586928 |
| Bak | 1.0 | 1.091768264570639 |
| Bim | 1.0 | 0.775572380916866 |
| Bik | 1.0 | 1.107008781595308 |
| Bcl-2 | 1.0 | 1.709214348529799 |
| Bcl-xL | 1.0 | 1.037659659159747 |
### Chart
| Category | LN18-Control | LN18-HRK |
|---|---|---|
| Bad | 1.0 | 2.302710960399964 |
| Bak | 1.0 | 1.827662900458798 |
| Bim | 1.0 | 1.697407942618245 |
| Bik | 1.0 | 0.14325592024625 |
| Bcl-2 | 1.0 | 0.480741526241326 |
| Bcl-xL | 1.0 | 0.716977624007914 |Gene expression
(fold of control)
### Chart
| Category | U87MG-Control | U87MG-HRK |
|---|---|---|
| Bad | 1.0 | 0.768437590644005 |
| Bak | 1.0 | 0.683020128377197 |
| Bim | 1.0 | 1.344124399593418 |
| Bik | 1.0 | 0.667419927085018 |
| Bcl-2 | 1.0 | 1.11987160404676 |
| Bcl-xL | 1.0 | 0.800144979722638 |Gene expression
(fold of control)
### Chart
| Category | U373-Control | U373-HRK |
|---|---|---|
| Bad | 1.0 | 1.262835451191641 |
| Bak | 1.0 | 0.939522749214012 |
| Bim | 1.0 | 1.037659659159746 |
| Bik | 1.0 | 0.801069877589622 |
| Bcl-2 | 1.0 | 1.344124399593415 |
| Bcl-xL | 1.0 | 0.954841603910418 |Gene expression
(fold of control)
Gene expression
(fold of control)
Supplementary Figure 1

## Slide 2
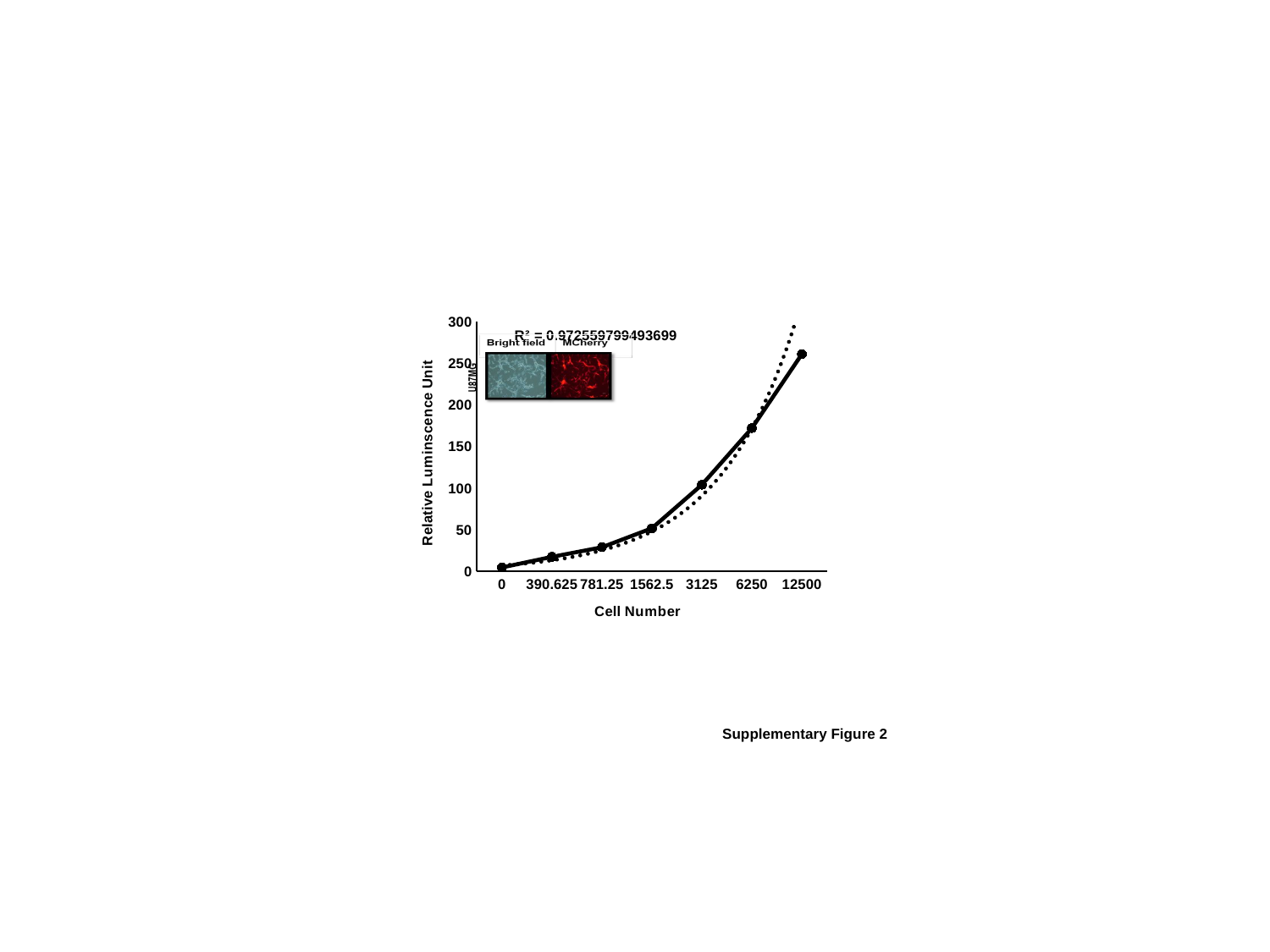

### Chart
| Category | |
|---|---|
| 0.0 | 4.75 |
| 390.625 | 17.5 |
| 781.25 | 29.0 |
| 1562.5 | 51.75 |
| 3125.0 | 104.0 |
| 6250.0 | 172.0 |
| 12500.0 | 260.75 |
Supplementary Figure 2

## Slide 3
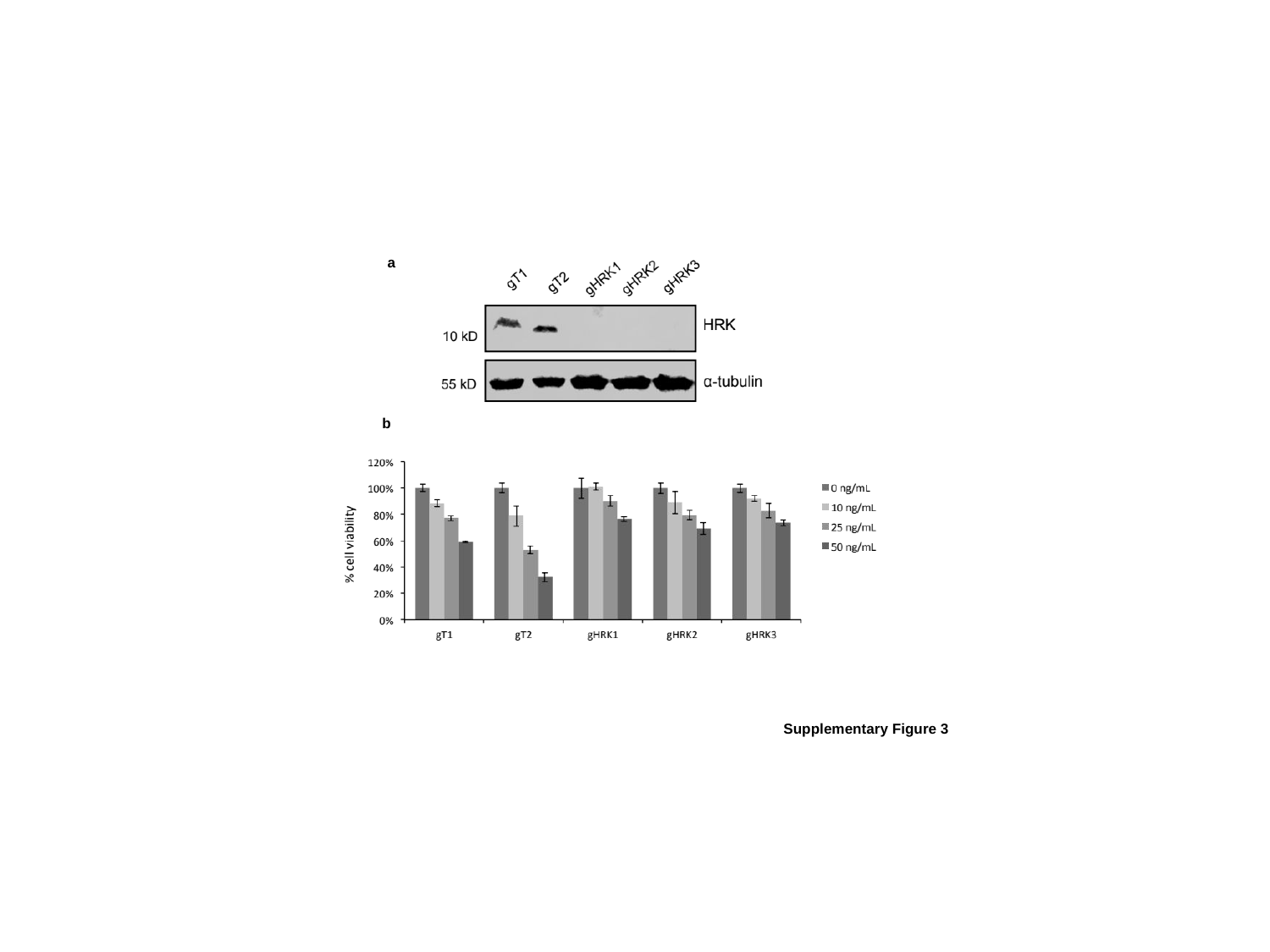

a
b
Supplementary Figure 3

## Slide 4
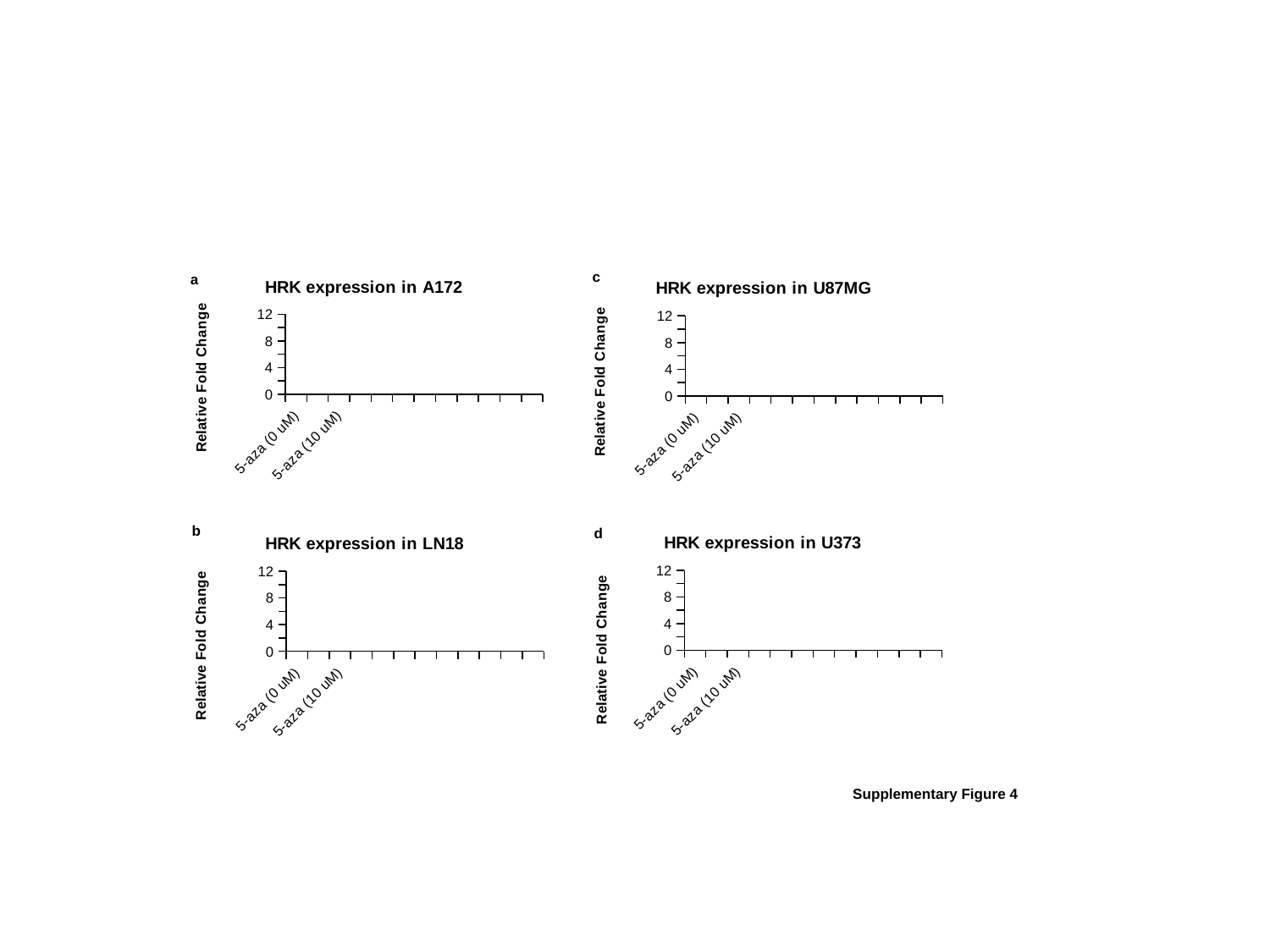

### Chart: HRK expression in A172
| Category | |
|---|---|
| 5-aza (0 uM) | 1.0 |
| 5-aza (2.5 uM) | 4.027822200226868 |
| 5-aza (10 uM) | 36.50443890778953 |c
### Chart: HRK expression in U87MG
| Category | |
|---|---|
| 5-aza (0 uM) | 1.0 |
| 5-aza (2.5 uM) | 2.114036081122765 |
| 5-aza (10 uM) | 11.47164198412663 |a
b
### Chart: HRK expression in U373
| Category | |
|---|---|
| 5-aza (0 uM) | 1.0 |
| 5-aza (2.5 uM) | 2.68514500556053 |
| 5-aza (10 uM) | 24.08394795857708 |
### Chart: HRK expression in LN18
| Category | |
|---|---|
| 5-aza (0 uM) | 1.0 |
| 5-aza (2.5 uM) | 9.546685944253769 |
| 5-aza (10 uM) | 78.79324245407447 |d
Supplementary Figure 4

## Slide 5
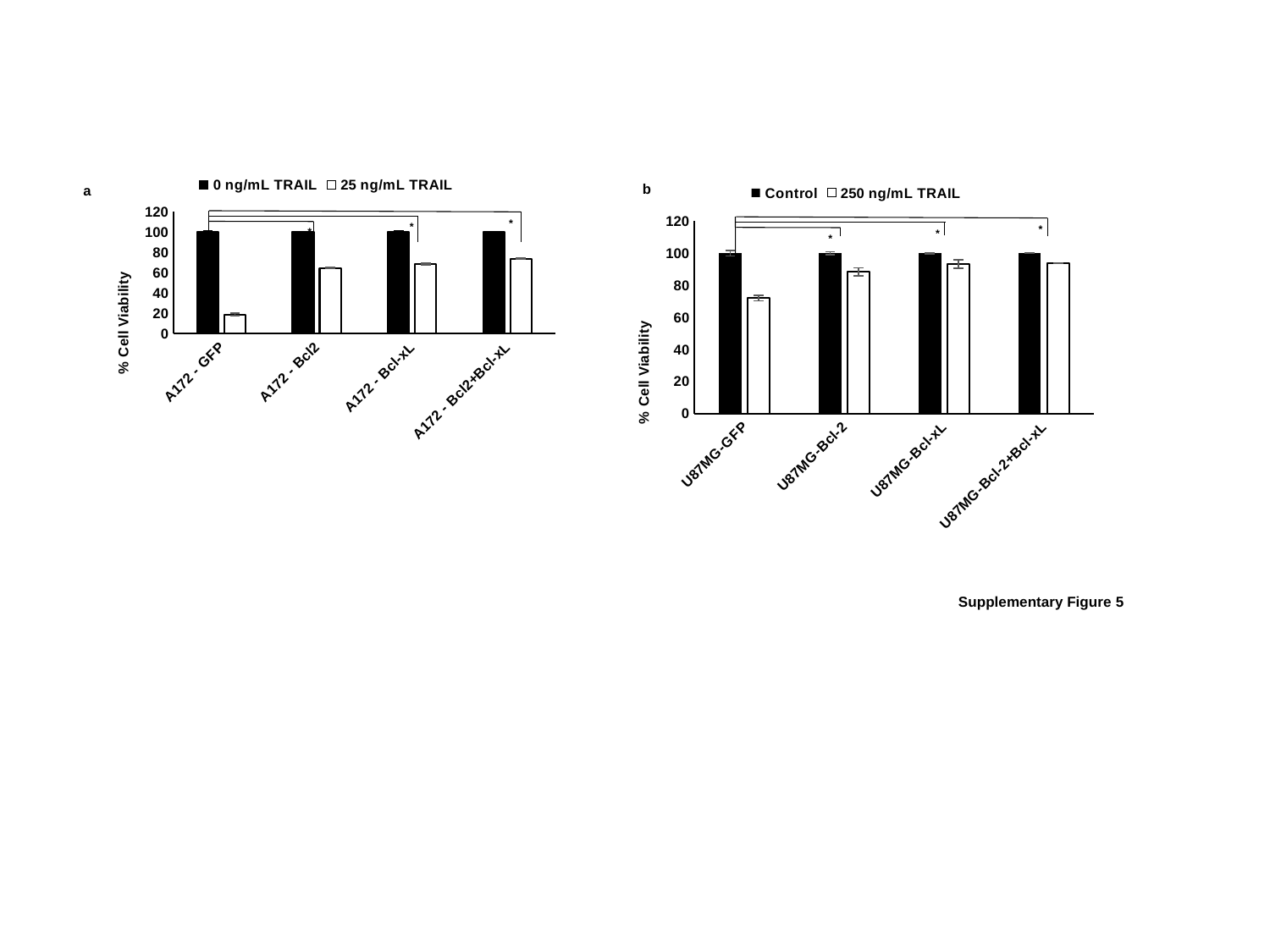

### Chart
| Category | 0 ng/mL TRAIL | 25 ng/mL TRAIL |
|---|---|---|
| A172 - GFP | 100.0 | 18.90938716005446 |
| A172 - Bcl2 | 100.0 | 64.3465956945053 |
| A172 - Bcl-xL | 100.0 | 68.4635290503256 |
| A172 - Bcl2+Bcl-xL | 100.0 | 73.69435343996916 |
### Chart
| Category | Control | 250 ng/mL TRAIL |
|---|---|---|
| U87MG-GFP | 100.0 | 71.99243718614755 |
| U87MG-Bcl-2 | 100.0 | 88.34746717374261 |
| U87MG-Bcl-xL | 100.0 | 93.22049294495245 |
| U87MG-Bcl-2+Bcl-xL | 100.0 | 93.6380419682679 |b
a
*
*
*
*
*
*
Supplementary Figure 5

## Slide 6
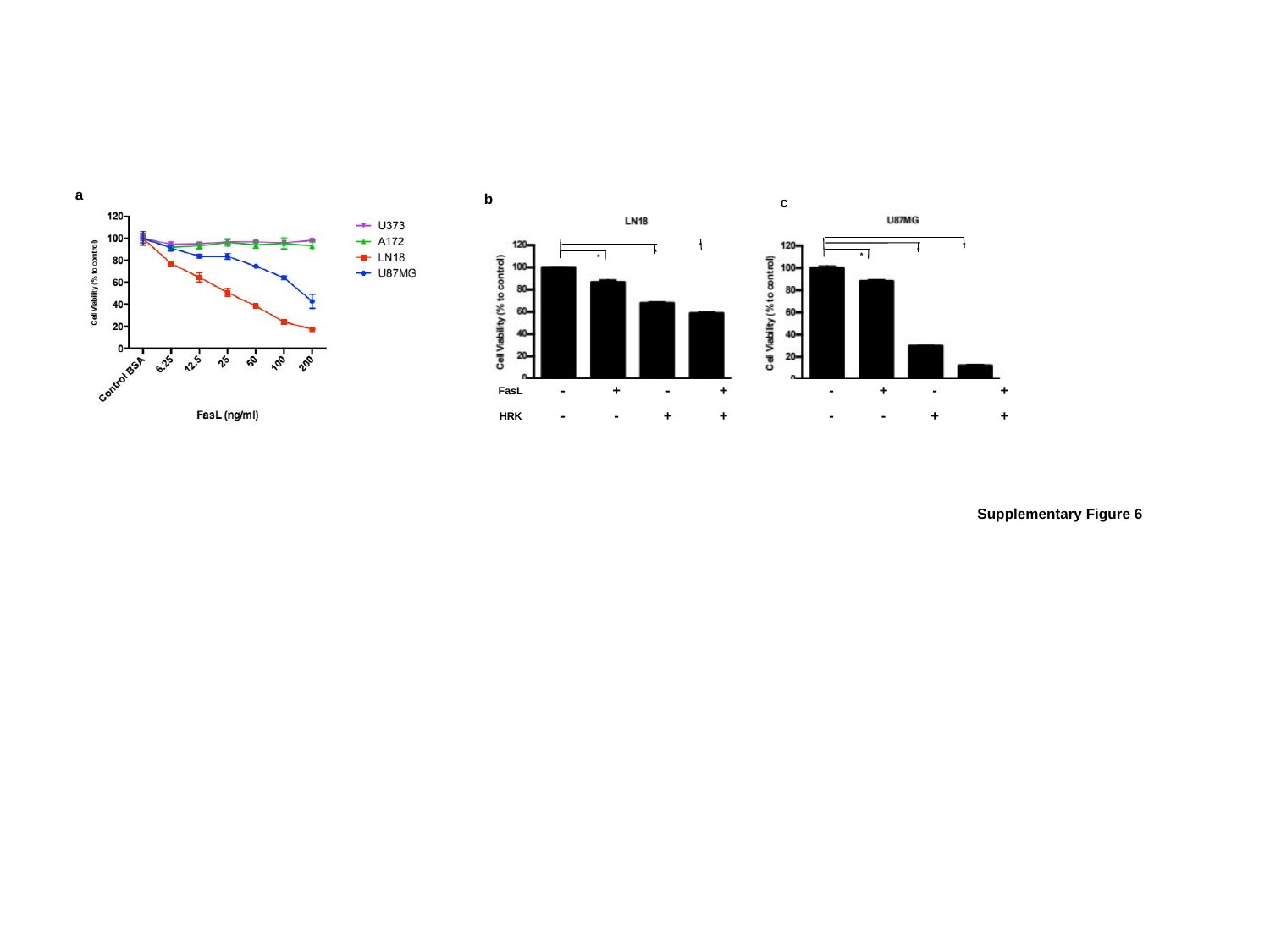

a
b
c
*
*
*
*
*
*
| FasL | - | + | - | + | | - | + | - | + |
| --- | --- | --- | --- | --- | --- | --- | --- | --- | --- |
| HRK | - | - | + | + | | - | - | + | + |
Supplementary Figure 6

## Slide 7
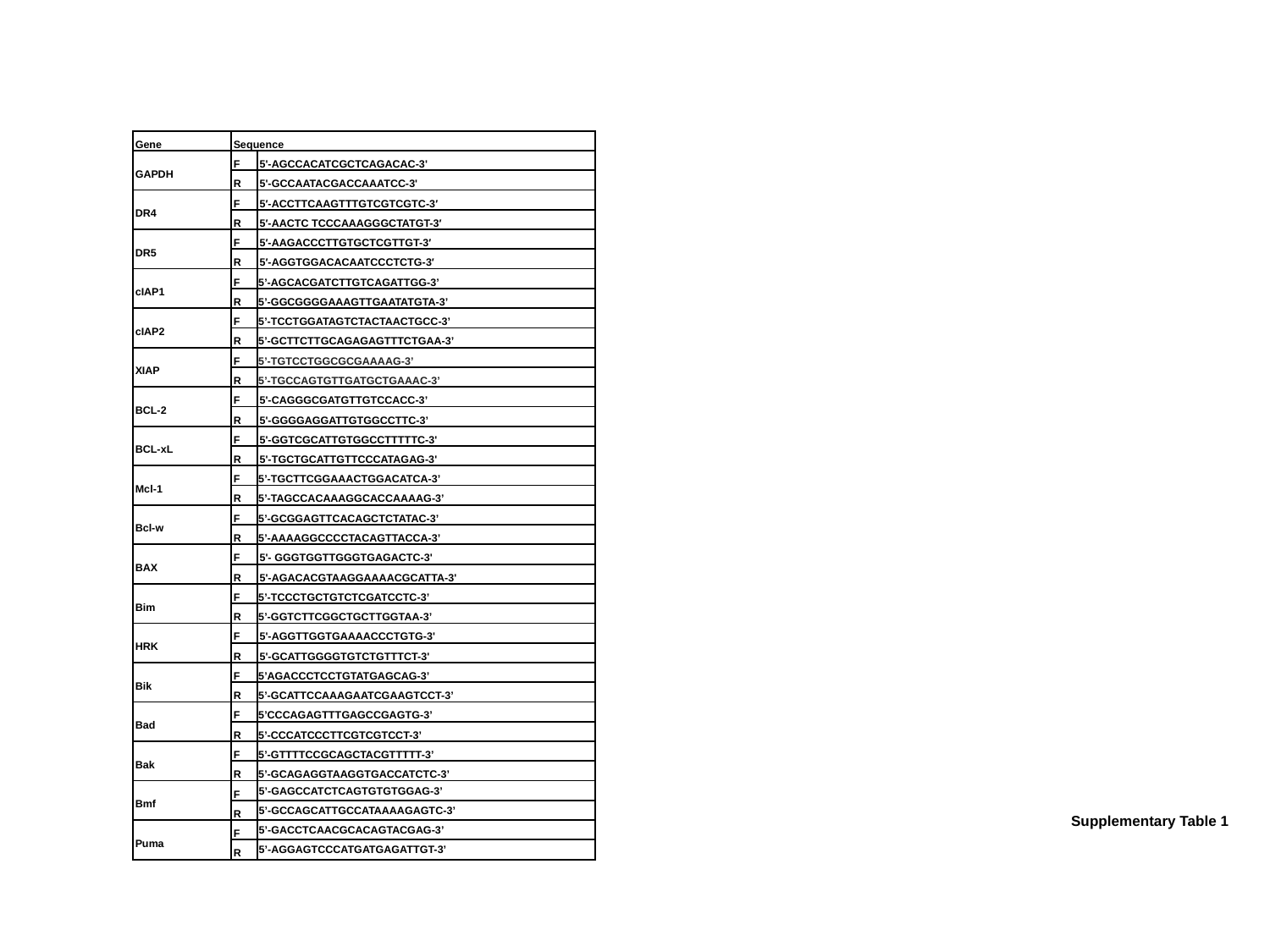

| Gene | Sequence | |
| --- | --- | --- |
| GAPDH | F | 5'-AGCCACATCGCTCAGACAC-3' |
| | R | 5'-GCCAATACGACCAAATCC-3' |
| DR4 | F | 5′-ACCTTCAAGTTTGTCGTCGTC-3′ |
| | R | 5′-AACTC TCCCAAAGGGCTATGT-3′ |
| DR5 | F | 5′-AAGACCCTTGTGCTCGTTGT-3′ |
| | R | 5′-AGGTGGACACAATCCCTCTG-3′ |
| cIAP1 | F | 5’-AGCACGATCTTGTCAGATTGG-3’ |
| | R | 5’-GGCGGGGAAAGTTGAATATGTA-3’ |
| cIAP2 | F | 5’-TCCTGGATAGTCTACTAACTGCC-3’ |
| | R | 5’-GCTTCTTGCAGAGAGTTTCTGAA-3’ |
| XIAP | F | 5’-TGTCCTGGCGCGAAAAG-3’ |
| | R | 5’-TGCCAGTGTTGATGCTGAAAC-3’ |
| BCL-2 | F | 5'-CAGGGCGATGTTGTCCACC-3’ |
| | R | 5'-GGGGAGGATTGTGGCCTTC-3’ |
| BCL-xL | F | 5'-GGTCGCATTGTGGCCTTTTTC-3' |
| | R | 5'-TGCTGCATTGTTCCCATAGAG-3' |
| Mcl-1 | F | 5’-TGCTTCGGAAACTGGACATCA-3’ |
| | R | 5’-TAGCCACAAAGGCACCAAAAG-3’ |
| Bcl-w | F | 5’-GCGGAGTTCACAGCTCTATAC-3’ |
| | R | 5’-AAAAGGCCCCTACAGTTACCA-3’ |
| BAX | F | 5'- GGGTGGTTGGGTGAGACTC-3' |
| | R | 5'-AGACACGTAAGGAAAACGCATTA-3' |
| Bim | F | 5’-TCCCTGCTGTCTCGATCCTC-3’ |
| | R | 5’-GGTCTTCGGCTGCTTGGTAA-3’ |
| HRK | F | 5'-AGGTTGGTGAAAACCCTGTG-3' |
| | R | 5'-GCATTGGGGTGTCTGTTTCT-3' |
| Bik | F | 5’AGACCCTCCTGTATGAGCAG-3’ |
| | R | 5’-GCATTCCAAAGAATCGAAGTCCT-3’ |
| Bad | F | 5’CCCAGAGTTTGAGCCGAGTG-3’ |
| | R | 5’-CCCATCCCTTCGTCGTCCT-3’ |
| Bak | F | 5’-GTTTTCCGCAGCTACGTTTTT-3’ |
| | R | 5’-GCAGAGGTAAGGTGACCATCTC-3’ |
| Bmf | F | 5’-GAGCCATCTCAGTGTGTGGAG-3’ |
| | R | 5’-GCCAGCATTGCCATAAAAGAGTC-3’ |
| Puma | F | 5’-GACCTCAACGCACAGTACGAG-3’ |
| | R | 5’-AGGAGTCCCATGATGAGATTGT-3’ |
Supplementary Table 1
